# The Saratoga Meeting of the American Medical Association—Notes by the Wayside

**Published:** 1902-08

**Authors:** H. A. West

**Affiliations:** Galveston, Texas


### Correspondence.

### The Saratoga Meeting of the American Medical Association—Notes by the Wayside.

BY H. A. WEST, M. D., GALVESTON, TEXAS.

*Editor Texas Medical Journal:*

It is due your readers who are members of the State Medical Association that as their delegate to the recent meeting of the American Medical Association I should make a report, especially upon those points which are of interest to them. I will attempt herein to present a cursory and informal statement.

I had to be at the meeting of the Secretaries of the various State medical associations, which had been called for the evening of the 9th of June, making it necessary for me to leave home on the 4th.

During the trip I was impressed more than ever with the greatness of this country. Passing through the waving grain fields of Texas, then ripening to the harvest, and acre after acre of corn and cotton at that time giving promise of an abundant yield, on through the beautiful scenery of the Ozark Mountains of Arkansas and Indian Territory; and through Missouri, with its extensive orchards and well-cultivated farms; through the great grain country east of St. Louis; through Illinois and Indiana; through thriving manufacturing towns; through the great natural gas and coal mining country; through the great and growing cities of Cleveland, Ohio, and Buffalo, New York; crossing that State along the banks of the classic Mohawk river, along the Erie canal; through a number of thriving and populous manufacturing towns of the Empire State. I was impressed more than ever with the magnitude of the agricultural and industrial interests of this grand country we live in, and with a realization to the fullest extent that it is the great granary and workshop of the world.

I remained over two days at Schenectady, and while there visited the General Electrical Works. Here I found a true industrial bee hive, with its ten thousand employes and one hundred thousand dollars per week pay roll. It is sufficient in itself to make and maintain a large city. It would be impossible for me to give an adequate idea of this factory. I found them filling orders for points in India, China and Japan, and just now an immense order is being completed to equip an electric system for the city of London, England.

I found Saratoga simply beautiful, and though straw hats were in evidence they looked a little out of season. It was so cold that I had to purchase an overcoat. The hotel accommodations were ample, the attendance was not as large as had been expected. The Secretaries' meeting was, I am sorry to say, slimly attended. I was honored by being elected chairman. We had several meetings. A full account of the details will be published in the *Journal of the American Medical Association.* We discussed plans of organization, and adjourned to meet next year at New Orleans. I trust that this may be the beginning of an important organization. It certainly seems that much good should be accomplished by meeting and consultation of these officers. Members who were at the Saratoga meeting could not fail to be impressed with the fine appearance of this body of men, representing as it does the very best material of the American medical profession.

Out of one hundred and fifty members of the House of Delegates only about ninety were present.

Judging from the details of published proceedings, you can understand that the members of this house had very little opportunity to attend the section meetings. Considering, however, that this was the first assembly of this body, and its method of proceeding had not been previously systemized, a large amount of work was accomplished, and with very little friction or discussion. Indeed, the mass of business requiring attention was so great that too little time could be allowed for proper discussion of many important matters. So far as the organization of the State Medical Associations was concerned, there was no dissent whatever to the plans of organization proposed by the committee composed of Drs. Simmons, McCormack and Foshay. And here I cannot refrain from expressing a word of appreciation for the excellence of the work accomplished by this committee, nor of the energy and wisdom displayed by them in its performance.

Dr. McCormack especially is an enthusiastic artist as an organizer. He informed me that in his county in Kentucky every physician was a member of the State and county society and of the American Medical Association. This is a simple illustration of what can be done elsewhere by properly directed efforts.

The operations of the House of Delegates were much facilitated by referring nearly all resolutions to a business committee; and the State of Texas, as well as myself, was honored by my appointment on this committee for the next year's meeting. I was also placed on the Committee on Public Health and on Transportation. For further details I refer the reader to the proceedings as published in the official journal. Judging from the first meeting, the House of Delegates may be considered as an immense success. Its accomplishments exemplified what can be done by similar bodies in the State societies. It is hoped that by next year by simplifying and condensing its *modus operandi* it can not only achieve more, but will be enabled to do so in good time, and allow its members an opportunity to attend sections.

I must give some account of the futile efforts to obtain the meeting of the American Medical Association for San Antonio. As it happened, I was placed upon the Nominating Committee, to select the place of meeting, and thus had a double responsibility—one to my constituents in Texas, and the other to the Association. I found the great difficulty in securing the meeting for San Antonio consisted in the prevailing doubts as to the ability of that city to provide suitable accommodations. When I learned that St. Paul and Minneapolis combined were considered insufficient and that Louisville, Kentucky, and Columbus, Ohio, were thought to be too small, I felt convinced that San Antonio's chances were very poor, indeed.

At the first meeting of the committee an informal ballot was taken, with the following results: Three for Asheville, N. C.; three for San Antonio, and two for Milwaukee. The next ballot resulted in four each for San Antonio and Asheville, Milwaukee being dropped.

It was then decided to postpone action until the afternoon. Upon another informal ballot Hot Springs came in with five votes to San Antonio's three. Finally Hot Springs was selected.

At this meeting I was enabled to introduce Dr. Paschal, feeling that he could present the claims of San Antonio better than myself. The only thing that brought a final defeat was the above mentioned opinion on the part of almost the entire committee, that San Antonio would not be able to care for the Association.

During the evening after the meeting adjourned the talk became general that New Orleans was in the field and that the report of the committee for Hot Springs would be rejected.

The next morning Dr. Gordon, of Maine, withdrew Hot Springs and substituted New Orleans, saying that he did it upon what he believed to be the unanimous conclusion of the committee. To this statement I dissented. However, New Orleans was accepted by a large majority vote on the part of those present.

It is perfectly useless for any city unable to afford accommodations for four to five thousand persons to make the attempt to secure the meeting. While the number of members does not begin to reach this figure, it is claimed that this number is made up by friends and families of physicians and of representatives of exhibitors.

Dr. John B. Murphy, the chairman of the committee, was an ardent friend of San Antonio. He suggested that an excursion to San Antonio and Mexico be offered. This, of course, will be left to the physicians and people of that city. At this time I cannot say whether it would be made a success or not. I simply write this to give the assurance that I left no effort untried in behalf of San Antonio.

In this connection it should be mentioned that the House of Delegates authorized the appointment of a paid organizer to assist in reorganization of the State societies. Dr. McCormack gave me his promise to be present at our meeting next year at San Antonio. Dr. Wyeth would also attend, but cannot do so on account of his expected visit to the International Medical Congress. He told me, however, that he would be present at one of the district association meetings.

I could not leave that part of the country without making a short visit to New York City, the great center of money and population, not only of this country, but of the world. I shall not attempt to describe the impression made upon me by the rush, push and power of this metropolis, where the people in the mad struggle for existence and wealth seem to jostle each other off the face of the earth.

I had time to visit only one hospital; that was the Post-Graduate. This is a model hospital in many respects, and to a great extent unique, its occupants being largely derived by a selection of material from an immense out-door clinic, especially for teaching purposes. While the number of beds is limited to about one hundred and fifty, yet in the variety and excellence of the clinical material it has hardly an equal.

I had the opportunity of attending the operative clinic of my younger brother, Dr. James N. West, Adjunct Professor of Gynecology of the Post-Graduate School, and will give brief notes of two interesting cases.

First, a New England maiden lady, age about forty, the subject of latent pulmonary tuberculosis. She had been suffering for several years with menorrhagia, uncontrollable by ordinary therapeutics. She had a double vagina and uterus. The operative measures required was incision of the vaginal septum with dilatation and curettement of the two uteri.

The first part of the procedure was particularly difficult on account of the extreme contraction of the vagina. As the operator, however, is an artist in this kind of work it was deftly and rapidly done. To control hemorrhage the line of incision was followed by a double row of continued catgut sutures. There was found to be two fully developed uteri, each with a separate cervix, body and canal. Cylindrical dilators were first used, followed by the use of the improved Goodell instrument. Irrigation and application of equal parts of carbolic acid and glycerine were done through a Polk's uterine speculum, which secures a much better result than the ordinary method. The customary packing of the uterus with iodiform gauze was dispensed with as superfluous. The operator, however, attaches great importance to the thorough application of the carbolized glycerine. The patient was progressing nicely at last accounts.

The second case was also a New England maiden, about forty years of age, and a sufferer from menorrhagia. An unruptured and dense hymen precluded an accurate diagnosis prior to the operation. Examination per rectum revealed a retroverted uterus and a small tumor whose relations could not be accurately defined. The uterus having been dilated, curretted and irrigated, then through an abdominal incision of about two and one-half inches the organ was drawn out and found to be the seat of two small fibroid tumors, which were eneucleated. The appendages were found to be healthy. The uterus was brought forward and stitched to the abdominal walls. The patient had hardly a rise of temperature, and no doubt progressed to rapid recovery.

I regret not having seen more of the operator's plastic work, as in this line he is recognized as without a superior in New York.

Galveston, July 15th.

